# The deubiquitinating enzyme MINDY2 promotes pancreatic cancer proliferation and metastasis by stabilizing ACTN4 expression and activating the PI3K/AKT/mTOR signaling pathway

**DOI:** 10.3389/fonc.2023.1169833

**Published:** 2023-05-03

**Authors:** Peng Liu, Songbai Liu, Changhao Zhu, Yongning Li, Ying Li, Xiaobin Fei, Junyi Hou, Xing Wang, Yaozhen Pan

**Affiliations:** ^1^ College of Clinical Medicine, Guizhou Medical University, Guiyang, China; ^2^ Department of Hepatic-Biliary-Pancreatic Surgery, The Affiliated Hospital of Guizhou Medical University, Guiyang, China; ^3^ Department of Hepatic-Biliary-Pancreatic Surgery, The Affiliated Cancer Hospital of Guizhou Medical University, Guiyang, China

**Keywords:** pancreatic cancer, MINDY2, deubiquitinating enzyme, ACTN4, PI3K/AKT/mTOR

## Abstract

The pathogenic mechanisms of pancreatic cancer (PC) are still not fully understood. Ubiquitination modifications have a crucial role in tumorigenesis and progression. Yet, the role of MINDY2, a member of the motif interacting with Ub-containing novel DUB family (MINDY), as a newly identified deubiquitinating enzyme, in PC is still unclear. In this study, we found that MINDY2 expression is elevated in PC tissue (clinical samples) and was associated with poor prognosis. We also found that MINDY2 is associated with pro-carcinogenic factors such as epithelial-mesenchymal transition (EMT), inflammatory response, and angiogenesis; the ROC curve suggested that MINDY2 has a high diagnostic value in PC. Immunological correlation analysis suggested that MINDY2 is deeply involved in immune cell infiltration in PC and is associated with immune checkpoint-related genes. *In vivo* and *in vitro* experiments further suggested that elevated MINDY2 promotes PC proliferation, invasive metastasis, and EMT. Meanwhile, actinin alpha 4 (ACTN4) was identified as a MINDY2-interacting protein by mass spectrometry and other experiments, and ACTN4 protein levels were significantly correlated with MINDY2 expression. The ubiquitination assay confirmed that MINDY2 stabilizes the ACTN4 protein level by deubiquitination. The pro-oncogenic effect of MINDY2 was significantly inhibited by silencing ACTN4. Bioinformatics Analysis and Western blot experiments further confirmed that MINDY2 stabilizes ACTN4 through deubiquitination and thus activates the PI3K/AKT/mTOR signaling pathway. In conclusion, we identified the oncogenic role and mechanism of MINDY2 in PC, suggesting that MINDY2 is a viable candidate gene for PC and may be a therapeutic target and critical prognostic indicator.

## 1 Introduction

Pancreatic cancer is a solid tumor of the gastrointestinal tract. It is the 11th most common cancer in women and the 12th most common cancer in men globally (1). Surgical techniques such as laparoscopic and robotic surgery are widely used to treat early-stage PC. Yet, patients with PC usually present with advanced-stage cancer at the time of diagnosis, thus losing their chance to undergo surgery (2–4). The lack of typical clinical symptoms and sensitive early diagnostic markers, coupled with the highly aggressive ability of PC, make its diagnosis and treatment very challenging (5). Consequently, it is necessary to investigate PC’s mechanism and identify valuable targets for early diagnosis and treatment.

Ubiquitination is an essential post-translational modification (PTM) that has a significant role in various aspects of the cellular life cycle, such as cell growth, proliferation, apoptosis, and DNA repair, especially in controlling substrate degradation and regulating protein “quality” and “quantity” (6, 7). Numerous studies have indicated that the loss of control of protein homeostasis leads to the development of many diseases, including tumors. Also, abnormalities in the ubiquitin-proteasome system (UPS) have been identified as an important cause of uncontrolled protein homeostasis. Moreover, it has been found that deubiquitinating enzymes (DUBs), as an essential component of the UPS, can remove the ubiquitin chain of protein substrates, thus reversing the ubiquitination process (8, 9). DUBs are involved in almost all cellular signaling pathways, such as gene transcription, cell cycle, and receptor downregulation, and abnormal DUBs have been associated with many diseases, especially tumors (10, 11), including pancreatic, lung, breast, and bladder cancers (12–19).

There are more than 100 DUBs in the human genome (20), which can be classified into seven families based on their catalytic mechanisms and structural similarities (21, 22), including ubiquitin carboxy-terminal hydrolases (UCHs), ovarian tumor proteases (OTUs), ubiquitin-specific proteases (USPs), Machado-Josephin domain-containing proteases (MJDs), MINDYs, JAB1/MPN/MOV34 metalloenzymes (JAMMs), and Zinc finger and UFSP structural domain protein (ZUFSP). Among them, the MINDY family is a recently discovered deubiquitinase family (23, 24). In this study, we examined the effect of MINDY2 (also known as FAM63B) in PC.

## 2 Results

### 2.1 MINDY2 is a potential oncogenic target for PC

**Figure 1:**
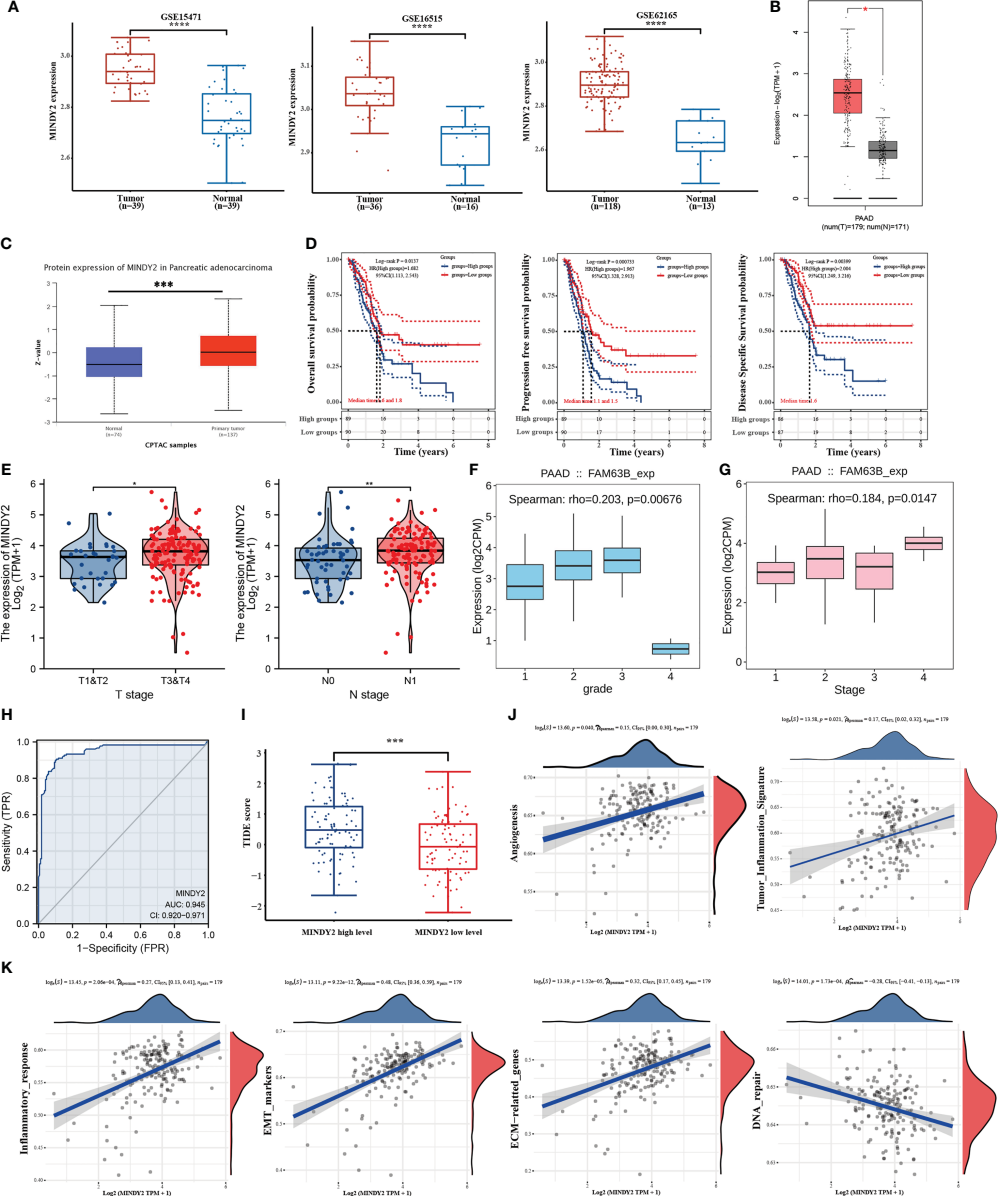
MINDY2 is a potential oncogenic target for PC. **(A, B)** Analysis of MINDY2 mRNA content in PC based on GEO and GEPIA2 datasets. **(C)** Analysis of MINDY2 expression levels of total protein in PC based on CPTAC dataset. **(D–G)** Correlation of MINDY2 with OS, PFS, DSS, T-stage, N-stage, tumor grade, and cancer stage in PC patients. **(H)** ROC curves to assess the diagnostic value of MINDY2 in PC. **(I)** TIDE algorithm to predict the relationship between MINDY2 and response to immunotherapy in PC. **(J, K)** Correlation of MINDY2 with EMT, inflammatory response, ECM-related genes, angiogenesis, tumor inflammatory features, and DNA repair. (*P < 0.05, **P < 0.01, ***P < 0.001, ****P < 0.0001).

To determine the potential function of MINDY2 in PC, we performed an analysis of three datasets from the GEO database (GSE15471, GSE16515, and GSE62165) as well as the GEPIA2 database. Higher expression of MINDY2 was found in PC tissues compared to adjacent normal tissues (**Figures 1A, B**). The CPTAC dataset in UALCAN revealed that the total protein expression level of MINDY2 was much higher in PC tissues than in adjacent normal tissues (**Figure 1C**).

Next, we obtained RNAseq data of PC from the TCGA database and corresponding clinical information and found an association between MINDY2 expression and overall survival (OS), progression-free survival (PFS), and disease-specific survival (DSS) (**Figure 1D**). Also, MINDY2 expression correlated with T-stage, N-stage, tumor grade, and cancer stage of PC (**Figures 1E–G**). The receiver operating characteristic (ROC) curve further suggested that MINDY2 has a high diagnostic value in PC (**Figure 1H**).

**Figure 2:**
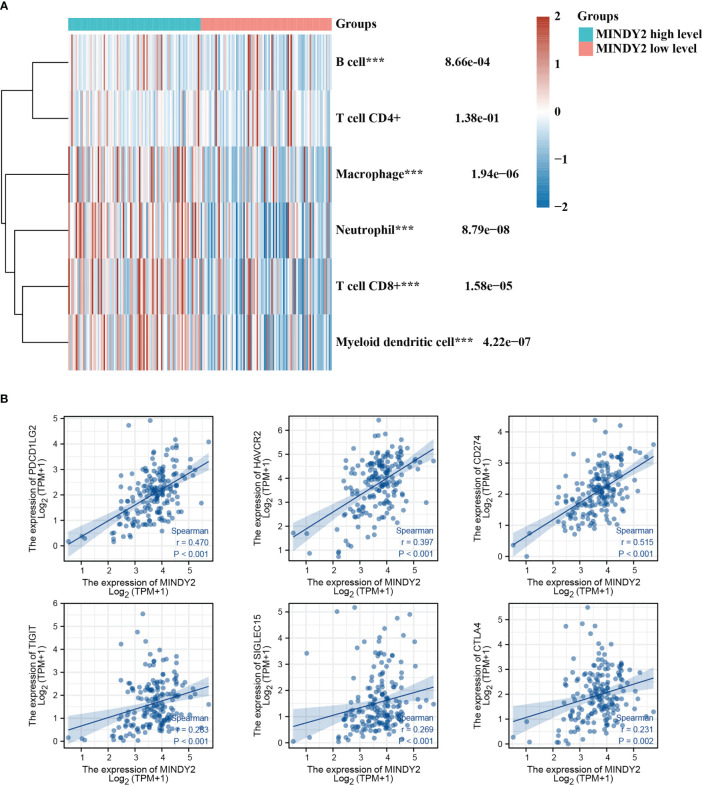
Correlation between MINDY2 and the score for tumor immune cell infiltration and immune checkpoint-related gene expression. **(A)** TIMER algorithm-based evaluation of the association between MINDY2 in PC and tumor immune cell infiltration score. **(B)** Correlation of MINDY2 in PC with immune checkpoint-related gene expression. (***P < 0.001).

The Tumor Immune Dysfunction and Exclusion (TIDE) algorithm predicted the immunotherapeutic response of MINDY2 in PC and discovered that the higher the expression of MINDY2, the better the response of PC to immune checkpoint inhibitors (**Figure 1I**). Then we further analyzed and found that MINDY2 was positively correlated with pro-cancer factors such as EMT, inflammatory response, ECM-related genes, angiogenesis, and tumor inflammatory features in PC, and negatively correlated with DNA repair capacity (**Figures 1J, K**). Also, immune correlation analysis revealed that the expression of MINDY2 in PC was positively correlated with the level of infiltration of B cells, T cells CD8+, neutrophils, macrophages, and dendritic cells (**Figure 2A**). Furthermore, correlation analysis between the expression of MINDY2 and the expression of immune checkpoint-related genes revealed that the expression of MINDY2 was correlated with PDCD1LG2, HAVCR2, CD274, TIGIT, and SIGLEC15 (**Figure 2B**). Therefore, the combined results of our bioinformatics analysis concluded that MINDY2 is a valuable oncogenic factor in PC.

### 2.2 MINDY2 expression is elevated in PC and is associated with poor prognosis

**Figure 3:**
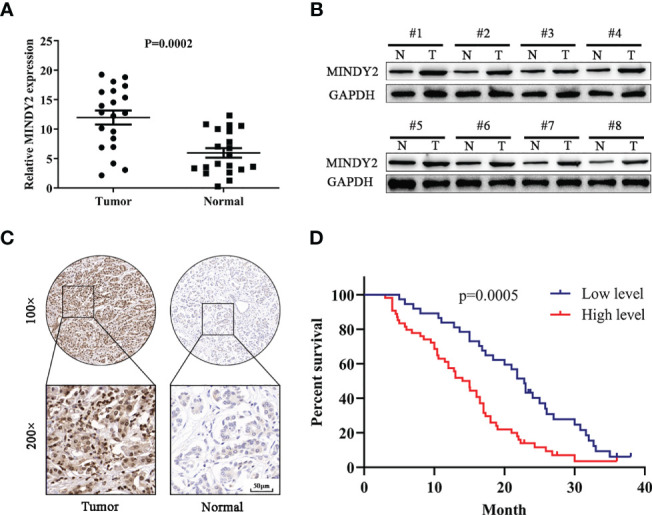
MINDY2 expression is elevated in PC and is associated with poor prognosis. **(A, B)** MINDY2 expression in PC tissues and adjacent normal tissues by Western blot and QT-PCR. **(C)** Immunohistochemical analysis of MINDY2 expression in TMA containing 90 PC and adjacent normal tissue. **(D)** Kaplan-Meier curve according to MINDY2 expression showing the survival rate of PC patients. Repre-sentative results of three biological replicates are shown.

We investigated the expression of MINDY2 in the cancerous and adjacent normal tissues of 20 PC patients. The results demonstrated that the mRNA and protein levels of MINDY2 expression were higher in cancer tissues than in nearby normal tissues (**Figures 3A, B**). Subsequently, we performed an immunohistochemical analysis in tissue microarrays (TMA) containing 90 PC and corresponding adjacent normal tissue samples. We discovered that the expression of MINDY2 was higher in PC tissues than in corresponding adjacent normal tissues (**Figure 3C**).

To further investigate the clinical significance of MINDY2, the relationship between MINDY2 expression and clinicopathological parameters of PC patients was examined. We discovered a correlation between higher MINDY2 and TNM stage, distant metastases, vascular invasion, and neurological invasion (**Table 1**). Moreover, the Kaplan-Meier analysis of survival demonstrated that the overall survival of patients with high MINDY2 expression was considerably lower than that of the control group (**Figure 3D**).

**Table 1:** Clinicopathological characteristics and the relationship between MINDY2 expression in PC patients.

			MINDY2 expression			P-value
Features		n	low	high	X^2^	
All cases		90	37	53		
Gender					0.118	0.828
Man		53	21	32		
Female		37	16	21		
Age					0.085	0.832
<60		47	20	27		
≥60		43	17	26		
pTNM stage					7.313	**0.009**
I and II		53	28	25		
III and IV		37	9	28		
Tumor size (cm)					2.295	0.174
<4		60	28	32		
≥4		30	9	21		
Lymph node metastasis					3.884	0.075
Negative		33	18	15		
Positive		57	19	38		
Distant metastasis					5.559	**0.028**
Negative		66	32	34		
Positive		24	5	19		
Perineural invasion					7.954	**0.007**
Negative		31	19	12		
Positive		59	18	41		
Blood vessel invasion					13.986	**0.000**
Negative		52	30	22		
Positive		38	7	31		

### 2.3 MINDY2 promotes PC cell proliferation, invasion, and migration *in vitro*

**Figure 4:**
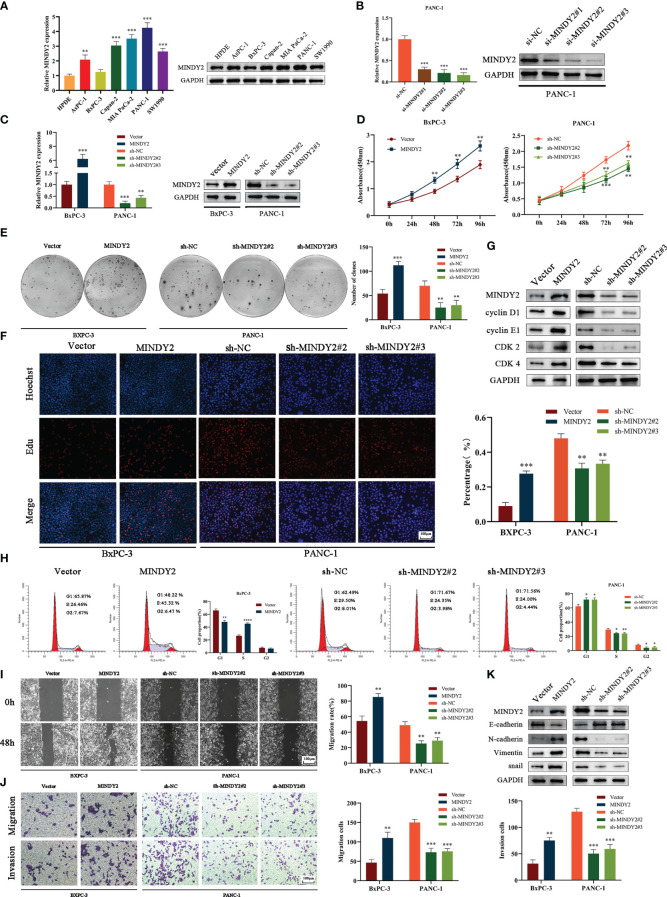
MINDY2 promotes PC cell proliferation, invasion, and migration *in vitro*. **(A)** The expression of MINDY2 in PC cell lines and HPDE detected using QT-PCR and Western blot. **(B)** QT-PCR and Western blot detected the silencing effect of three small interfering RNA sequences. **(C)** QT-PCR and Western blot assay for stable up/down-regulation of lentivirus transfection efficiency. **(D)** The effect of MINDY2 on PC cell viability assessed by CCK8 assay. **(E)** Cloning plate assay to estimate the influence of MINDY2 on the ability of PC cells to form clones. **(F)** Edu assay to evaluate the influence of MINDY2 on the proliferative capacity of PC cells. **(G)** Western blot to detect the effect of MINDY2 on cell cycle proteins. **(H)** Flow cytometry to analyze the impact of MINDY2 on the cell cycle in PC cells. **(I, J)** To evaluate the impact of MINDY2 on PC cells’ capacity for invasion and migration, wound healing assays and Transwell assays were conducted. **(K)** Western blot detection of MINDY2’s effect on EMT. (*P < 0.05, **P < 0.01, ***P < 0.001, ****P < 0.0001). Repre-sentative results of three biological replicates are shown.

To further investigate the biological function of MINDY2 in PC, we discovered that the expression of MINDY2 was higher in PC cell lines (AsPC-1, BxPC-3, Capan-2, PANC-1, Mia PaCa-2, and SW1990) than in normal pancreatic ductal epithelial cells (HPDE); the lowest endogenous expression level of MINDY2 was seen in BxPC-3 cells and the highest expression in PANC-1 cells (**Figure 4A**). Therefore, BxPC-3 and PANC-1 were used as the target cells for this study.

Next, we designed 3 small interfering RNAs and validated their efficiency in PANC-1 cells. The results suggested that si-MINDY2#2 and si-MINDY2#3 sequences had the best silencing effect (**Figure 4B**). Thus, si-MINDY2#2 and si-MINDY2#3 sequences were used to construct stable up/down-regulated lentiviral vectors for target cell infection and to verify the infection efficiency (**Figure 4C**). The cell viability and colony-forming ability of PC cells were significantly reduced after silencing MINDY2, and this ability was significantly enhanced after upregulating MINDY2 by CCK8 and clonogenic plate experiments (**Figures 4D, E**). EdU assay demonstrated that the proliferation ability of BxPC-3 cells was significantly increased after MINDY2 overexpression compared with the diminished proliferation capacity of PANC-1 cells after the down-regulation of MINDY2 (**Figure 4F**).

Western blot analysis revealed that the expression levels of Cyclin D1, Cyclin E1, CDK2, and CDK4 increased following MINDY2 overexpression, while these cyclins were decreased after the downregulation of MINDY2 compared with the control group (**Figure 4G**). In addition, flow cytometry showed that the G1 phase of BxPC-3 cells decreased significantly after overexpression of MINDY2. In contrast, the S and G2 phases significantly increased, which resulted in an accelerated G1/S phase transition of the cell cycle, while the results were reversed after the downregulation of MINDY2 (**Figure 4H**). Thus, elevated expression of MINDY2 promoted the proliferation of PC cells.

Wound healing assay and Transwell assay revealed that PC cells had greater vital invasive and migratory ability after upregulation of MINDY2. In contrast, this ability was significantly reduced after the knockdown of MINDY2 (**Figures 4I, J**). The identification of EMT-related proteins by Western blot revealed that the MINDY2-overexpressed group expressed higher levels of N-cadherin, Snail, and Vimentin than the control group. The levels of E-cadherin protein expression were lower than in the control group. Opposite results were obtained after the downregulation of MINDY2 (**Figure 4K**). In light of the preceding findings, MINDY2 may increase the invasion and metastasis of PC cells *via* EMT.

### 2.4 MINDY2 promotes PC growth and liver metastasis *in vivo*

**Figure 5:**
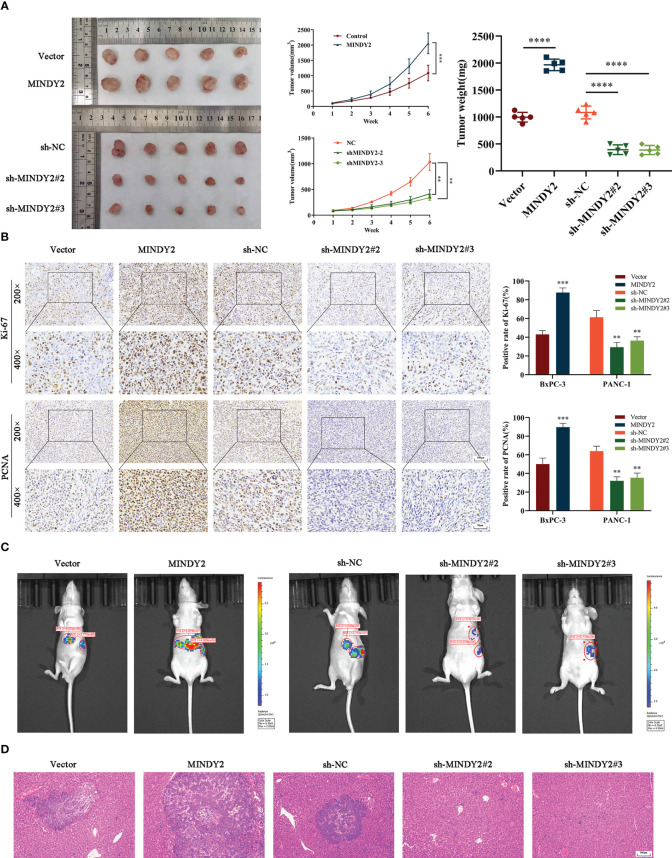
MINDY2 promotes PC growth and liver metastasis *in vivo*. **(A)** A nude mouse xenograft model was constructed to observe the effect of MINDY2 on the subcutaneous tumorigenic ability of nude mice. **(B)** IHC observed Ki67 and PCNA expression levels in tumor tissues. **(C)** Construction of a splenic envelope liver metastasis model in order to examine the effect of MIDNY2 on the ability of liver metastasis.**(D)** Representative HE stained images of liver metastases. (*P < 0.05, **P < 0.01, ***P < 0.001, ****P < 0.0001). Five repetitions were set for each group.

To further investigate the effect of MINDY2 in tumors, we subcutaneously injected different groups of PC cells into the right axilla of nude mice to evaluate the effect of MINDY2 on tumor growth. The results showed that the volume and weight of tumor formation in nude mice were significantly higher than those in the control group after the upregulation of MINDY2; opposite result was obtained after the downregulation of MINDY2 (**Figure 5A**). In addition, the immunohistochemical results of tumor tissue showed that after the stable up-regulation of MINDY2, the staining intensity and positive ratio of MINDY2 in tumor tissue were higher than those in the control group. The staining of PCNA and Ki67 related to proliferation indicators was also enhanced, and the positive ratio was significantly higher than that in the control group; opposite results were seen after downregulation of MINDY2 (**Figure 5B**).

Meanwhile, we implanted PC cell suspensions with stable upregulation of MINDY2 in the spleen of nude mice to construct a liver metastasis model. We found that the size and number of liver metastases were significantly more substantial in the upregulated group than in the control group. In contrast, the knockdown MINDY2 group significantly inhibited PC cell liver metastasis *in vivo* (**Figure 5C**). HE staining of mouse liver tissue also obtained the same result (**Figure 5D**). Thus, MINDY2 promoted PC growth and liver metastasis *in vivo*.

### 2.5 MINDY2 interacts with ACTN4 and stabilizes ACTN4 by deubiquitination function

**Figure 6:**
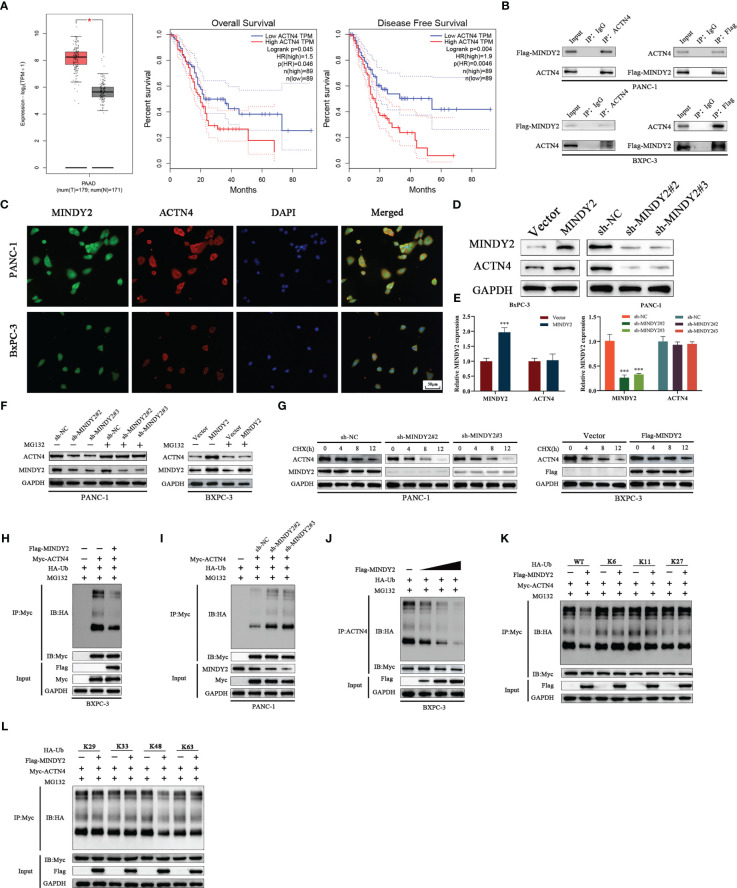
MINDY2 interacts with ACTN4 and stabilizes ACTN4 by deubiquitination function. **(A)** The expression level of ACTN4 in PC and the relationship with OS and DFS were analyzed based on the GEPIA2 database. **(B)** Immunoprecipitation experiments to detect the interaction between MINDY2 and ACTN4. **(C)** Immunolocalization assay to detect the effect of MINDY2 and ACTN4 in the cell. **(D, E)** Western blot and QT-PCR were utilized to detect the effect of MINDY2 on the protein and mRNA expression of ACTN4. **(F)** Western blot to determine the impact of MINDY2 on ACTN4 protein levels in cells treated with MG132. **(G)** CHX treatment of cells to observe the effect of MINDY2 on ACTN4 half-life. **(H, I)** Western blot examined the ubiquitination level of ACTN4 in Flag-MINDY2, HA-Ub and Myc-ACTN4 cotransfected BxPC-3 cells. **(J)** MINDY2 regulates the ubiquitination level of ACTN4 in a dose-dependent manner. **(K, L)** Western blot detection of ubiquitination of ACTN4 in Flag-MINDY2, Myc-ACTN4 and HA-Ub mutants (HA-WT, K6, K11, K27, K29, K33, K48 or K63) cotransfected BxPC-3 cells. (*P < 0.05, **P < 0.01, ***P < 0.001). Repre-sentative results of three biological replicates are shown.

To further investigate the mechanism of MINDY2 in PC, we performed protein profiling on the samples obtained from MINDY2 immunoprecipitation experiments. ACTN4 was identified as the target protein of MINDY2. According to bioinformatics analyses, ACTN4 is significantly expressed in PC and is associated with a bad prognosis (**Figure 6A**, **Supplementary Figure 1**). Immuno-co-precipitation experiments further showed that MINDY2 interacted with ACTN4 (**Figure 6B**). In addition, immunofluorescence co-localization revealed that MINDY2 and ACTN4 were mainly co-localized in the cytoplasm in PC cells (**Figure 6C**).

Western blot assay suggested that MINDY2 could regulate the level of ACTN4 protein (**Figure 6D**). However, QT-PCR results indicated that MINDY2 does not impact the mRNA expression of ACTN4 (**Figure 6E**). We also discovered that the addition of the proteasome inhibitor MG132 eliminated MINDY2’s ability to regulate ACTN4 protein expression (**Figure 6F**). Then, we used the protein synthesis inhibitor actinomycin (CHX) to further show how MINDY2 affects the stability of the ACTN4 protein. The ACTN4 half-life was significantly shorter in cells that down-regulated MINDY2 and significantly longer in cells that overexpressed MINDY2 (**Figure 6G**). The results suggest that MINDY2 may regulate ACTN4 protein amount through deubiquitination modifications. To further confirm this conjecture, we performed ubiquitination experiments to analyze whether ACTN4 is a deubiquitinated substrate for MINDY2. Ubiquitination experiments showed that over-expression of MINDY2 markedly decreased the ubiquitination level of ACTN4; at the same time, the level of ACTN4 ubiquitination was significantly higher after MINDY2 downregulation (**Figures 6H, I**).

Moreover, the protein levels of ACTN4 and the ubiquitination level were dose-dependent for MINDY2 (**Figure 6J**). To further investigate the effect of MINDY2 on the type of deubiquitination modification of ACTN4, we co-expressed Flag-MINDY2 and Myc-ACTN4 as well as mutant HA-ub in BxPC-3 cells. The ubiquitination experiments showed that MINDY2 cleaved only the K48 ubiquitin chain on ACTN4 (**Figures 6K, L**). Thus, the above results suggested that MINDY2 stabilizes ACTN4 protein expression by cleaving the K48 chain linked to ACTN4 to avoid its degradation by ubiquitination.

### 2.6 MINDY2 activates the PI3K/AKT/mTOR pathway by stabilizing ACTN4

**Figure 7:**
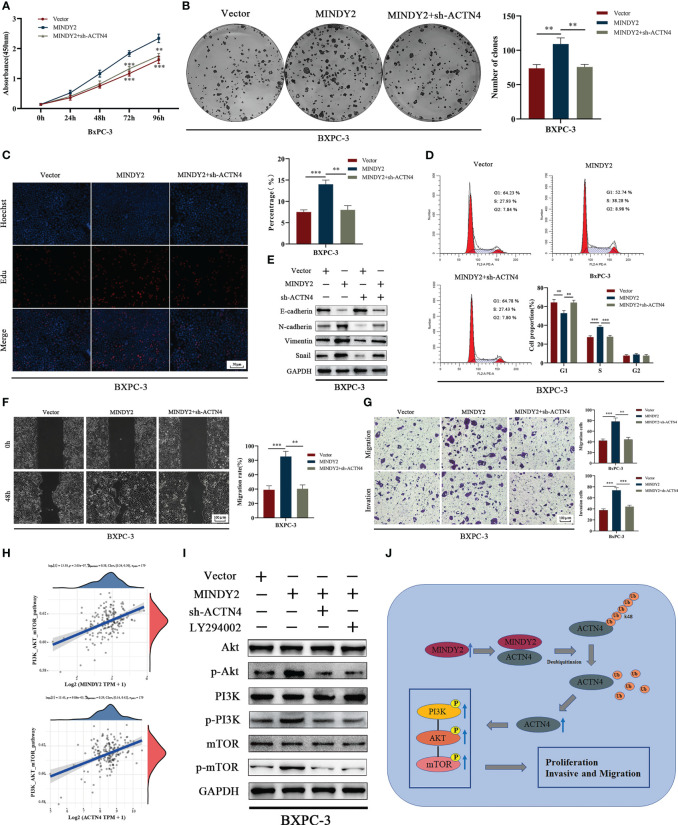
MINDY2 stabilizes ACTN4 to activate the PI3K/AKT/mTOR pathway, which promotes cancer development in PC. **(A–D)** CCK8, clonogenic plate assay, Edu, and flow cytometry to detect the effect of silencing ACTN4 on the ability of MINDY2 to promote PC cell proliferation. **(E)** Western blot to detect the effect of silencing ACTN4 on MINDY2 to promote EMT function. **(F, G)** After silencing ACTN4, a wound-healing experiment and a Transwell assay were conducted to determine the effect of MINDY2 on PC cell invasion and migration. **(H)** TCGA database-based analysis of the relationship between MINDY2, ACTN4, and PI3K/AKT/mTOR pathway. **(I)** In PC cells overexpressing MINDY2, Western blot analysis revealed alterations in the total protein levels and phosphorylation levels of PI3K, AKT, and mTOR after silencing ACTN4 or adding the PI3K inhibitor LY294002. **(J)** Mechanistic model of the MINDY2-ACTN4-PI3K/AKT/mTOR pathway axis in PC. (** P < 0.01, *** P < 0.001). Repre-sentative results of three biological replicates are shown.

To further clarify the role of ACTN4 in the cancer-promoting process of MINDY2, after we down-regulated ACTN4 and upregulated MINDY2 co-treated BxPC-3 cells, the results of CCK-8, clonal plate, and EdU experiments suggested that the function of MINDY2 in enhancing PC cell viability and proliferation ability was reversed (**Figures 7A–C**). Flow cytometry demonstrated that MINDY2 inhibited the G1/S phase transition of the cell cycle (**Figure 7D**). Also, it was found that the knockdown of ACTN4 reduced the ability of MINDY2 to promote EMT (**Figure 7E**); both the Transwell test and the wound healing experiment demonstrated that MINDY2’s capacity to encourage PC cell invasion and migration was significantly reduced (**Figures 7F, G**).

The PI3K/AKT/mTOR signaling pathway is essential for PC development (25–28), and ACTN4 performs a pro-cancer role in malignancies by activating this pathway (29, 30). Bioinformatics analysis indicted that both MINDY2 and ACTN4 in PC were associated with the PI3K/AKT/mTOR signaling pathway (**Figure 7H**). After MINDY2 overexpression, the total protein levels of PI3K, AKT, and mTOR did not change. In contrast, the phosphorylation level was significantly increased, and the addition of PI3K inhibitor LY294002 reversed this function of MINDY2, A similar effect was obtained after silencing ACTN4 (**Figure 7I**). Thus, ACTN4 is crucial in the activation of the PI3K/AKT/mTOR signaling pathway by MINDY2. According to the aforementioned findings, MINDY2 stabilizes ACTN4 expression by deubiquitinating it, which then stimulates the PI3K/AKT/mTOR pathway to encourage PC proliferation and invasive metastasis (**Figure 7J**).

## 3 Discussion

Ubiquitination is a very important PTM that has a vital role in the degradation of proteins and in maintaining intracellular environmental homeostasis (31). Three enzymes-ubiquitin activating enzymes (E1), ubiquitin-binding enzyme (E2), and ubiquitin ligase (E3)—are primarily responsible for mediating the ubiquitination process (32); yet, ubiquitination is a reversible process. For example, E3 ubiquitin ligase selectively mediates the ubiquitin-binding of substrates, while DUB negatively regulates this process so that ubiquitination and deubiquitination are maintained in dynamic balance under the coordinated action of E3 ubiquitin ligase and DUB (33). Recent investigations have demonstrated that DUB is crucial for the development of tumors, and MINDY2, a recently discovered deubiquitinating enzyme, has a unique selectivity for cleaving K48-linked polyUb (34). Nevertheless, the biological function of MINDY2 in PC has not been studied.

In this study, we first performed bioinformatics analysis by GEPIA2, GEO, and TCGA databases. We found that MINDY2 was expressed elevated in PC at mRNA and protein levels and correlated with prognostic indicators such as OS, PFS, DSS, T stage, N stage, tumor grading, and tumor stage in patients. In addition, the ROC curve indicated that MINDY2 had a high diagnostic value for PC.

In recent years, tumor immunotherapy has become a hot spot in the field of tumor treatment research and is also considered one of the most promising treatment methods. Immunotherapy has achieved great success in the treatment of many tumors (35); however, its therapeutic effect in PC is minimal (36). In this study, we analyzed the correlation between MINDY2 in PC and immunity and found that MINDY2 was significantly and positively correlated with infiltration scores of B cells, T cells CD8+, neutrophil, macrophage, and myeloid dendritic cells in PC, and although there was no statistical significance between MINDY2 and T cells CD4+, it can be concluded that the expression level of CD4+ T cells was greater in the MINDY2 high expression group than in the low expression group.

The immune checkpoint acts as an immune system suppressor, which prevents the body’s immune system from producing an effective anti-tumor immune response by suppressing immune cell function, hence facilitating the immune evasion of the tumor (37). Therefore, we analyzed the relationship between MINDY2 expression and immune checkpoint-related genes in PC, finding a strong and positive correlation between MINDY2 expression and PDCD1LG2, HAVCR2, CD274, TIGIT, SIGLEC15, and CTLA. In addition, the TIDE algorithm indicated that a higher expression of MINDY2 was correlated with a better response of PC to immune checkpoint inhibitors. Then, we found that MINDY2 was associated with PC tumorigenic factors such as EMT, inflammatory response, and ECM-related genes. Therefore, based on the results of bioinformatics analysis, we have reason to believe that MINDY2 is an oncogene with significant value in PC.

To further elucidate the biological functions of MINDY2 in PC, we collected clinical samples for assay. We found that the expression level of MINDY2 was elevated in PC. *In vitro* experiments, including cloning plate, CCK-8 assay, wound healing assay, and transwell assay, revealed that MINDY2 overexpression was associated with a higher rate of PC proliferation and migration; this process was decreased after the knockdown of MINDY2. We then confirmed this ability of MINDY2 in Western blot assays to detect cell cycle-associated proteins, flow cytometry to detect cell cycle, subcutaneous tumorigenesis in nude mice, and splenic envelope liver metastasis. Activation of EMT leads to changes in cell migration and invasive functions (38, 39). Previous studies also found that the metastasis of PC is associated with EMT (40). Therefore, we detected the EMT-related protein expression and discovered that upregulation of MINDY2 was followed by upregulation of N-cadherin, Snail, and vimentin and downregulation of E-cadherin, while downregulation of MINDY2 reversed this process. Therefore, we speculate that MINDY4 in PC enhances the invasion and migration of PC by inducing EMT.

To further investigate the cancer-promoting mechanism of MINDY2 in PC, we identified ACTN4 as an interacting protein of MINDY2 by immunoprecipitation, mass spectrometry, and immunofluorescence co-localization. Notably, ACTN4 protein levels increased after overexpression of MINDY2, while mRNA levels did not significantly change, suggesting that MINDY2 is not an upstream transcription factor of ACTN4 but regulates the changes of ACTN4 through PTM. Next, we investigated whether MINDY2, a member of the deubiquitinating enzyme family, can modify ACTN4 by deubiquitination. First, we found that the half-life of ACTN4 was significantly prolonged after treating cells overexpressing MINDY2 with CHX, and the half-life of ACTN4 was shortened after silencing MINDY2.

Furthermore, the regulatory effect of MINDY2 on ACTN4 protein disappeared after the addition of MG132-treated cells. Hence, we speculate that MINDY2 stabilizes ACTN4 protein expression through deubiquitination. To further explore the regulatory mechanism, we conducted ubiquitination experiments on BxPC-3 cells after transfecting them with ubiquitin. We discovered that MINDY2 might, by explicitly deleting the K48-linked ubiquitin chain on ACTN4, lower the degree of ubiquitination of ACTN4 and block proteasome-mediated degradation of ACTN4 protein. Therefore, we concluded that MINDY2 stabilizes ACTN4 protein expression through deubiquitination. However, previous research has demonstrated that ACTN4 is crucial for carcinogenesis, metastasis, and EMT (41, 42), so it was unclear whether the pro-carcinogenic role of MINDY2 in PC is related to ACTN4. Therefore, we conducted a series of cell biology tests after silencing ACTN4 in PC cells overexpressing MINDY2 and discovered that silencing ACTN4 reversed the pro-carcinogenic role of MINDY2 in PC. Thus, ACTN4 is essential in the cancer-promoting process of MINDY2. Moreover, bioinformatics analysis found that both MINDY2 and ACTN4 were correlated with the PI3K/AKT/mTOR signaling pathway in PC. In addition, Western blot found that the phosphorylation levels of PI3K, AKT, and mTOR were increased after MINDY2 overexpression without significant changes in protein levels, and the addition of PI3K inhibitor LY294002 reversed this function of MINDY2; a similar effect was obtained after silencing ACTN4. Thus, ACTN4 is essential in the activation of the PI3K/AKT/mTOR signaling pathway by MINDY2.

## 4 Conclusion

we discovered that MINDY2 stabilizes ACTN4 protein expression through the deubiquitination function in PC, which activates PI3K/AKT/mTOR signaling pathway and promotes PC proliferation, invasion, and migration. Although we still have not cracked the therapeutic code of PC, targeting MINDY2 may provide new hope for the treatment of PC.

## 5 Materials and methods

### 5.1 Bioinformatics analysis

Bioinformatics analysis of three datasets (GSE15471, GSE16515, and GSE62165) was obtained in the Gene Expression Omnibus (GEO) database (https://www.ncbi.nlm.nih.gov/geo/); patients undergoing chemotherapy or radiotherapy were excluded. The data were processed using the “Limma” package in Rstudio, and box line plots were plotted by a boxplot. Gene Expression Profiling Interactive Analysis 2 (GEPIA2, https://GEPIA2.cancerpku.cn/35; general) is an analysis of gene expression in cancer and non-cancer tissues based on The Cancer Genome Atlas (TCGA, https://cancergenome.nih.gov) and Genotype-tissue Expression (GTEx). On this website, we obtained box-line plots of expression differences between PC and normal tissue in the GTEx database (43).

The CPTAC (Clinical Proteomic Tumor Analysis Consortium) dataset of UALCAN (https://UALCAN.path.uab.edu/analysisprot.html) was utilized to examine protein expression differences between PC and normal tissues, and a Box line graph was generated. The TCGA database provided the RNAseq data (level 3) for PC and the related clinical data, utilizing the “survminer” package in R software for visualization and the “survivor” package for statistical analysis of survival data. The analysis of the T-stage and M-stage was visualized using the “ggplot2” package. An immune assessment was performed with the “immunedeconv” package of R software; a reliable R package was used for immune assessment, using the TIMER algorithm to evaluate the connection between MINDY2 expression and six immune cell infiltration scores. “ggplot2” and “pheatmap” were used to analyze and generate heat maps. The relationship between MINDY2 expression and immune checkpoint-related genes in PC was analyzed and visualized by the “ggplot2” package. The TIDE algorithm was used to predict the potential association between MINDY2 and PC response to immunotherapy, plotted and analyzed using the “ggplot2” and “ggpubr” packages of R software (44). The genes found in the associated pathways were then gathered and analyzed using the R software package “GSVA” with the parameter method= “ssgsea.” The correlation between the scores of the genes and pathways was examined using Spearman’s correlation.

In the TISIDB database (http://cis.hku.hk/TISIDB/), we searched for correlations between MINDY2 expression and tumor grade and cancer stage. This database combines a variety of data sources to investigate interactions between the immune system and tumors (45). Regarding ROC curves, RNAseq and relevant clinical data for PCs were obtained from the TCGA and GTEx databases. Statistical analysis and visualization were performed with R v4.2.1 software (analysis with the “pROC” package and data visualization with the “ggplot2” package) (46). The horizontal and vertical coordinates are the false positive rate (FPR) and the actual positive rate (TPR), respectively (the ROC curve’s area under the curve ranges from 0.5 to 1, and diagnostic accuracy increases when the AUC is close to 1. When the AUC is between 0.5 and 0.7, accuracy is poor; when it is between 0.7 and 0.9, accuracy is moderate; and when it is beyond 0.9, accuracy is high).

### 5.2 Human tissue samples

Twenty cases of fresh PC tissues and adjacent normal tissues were acquired from the Affiliated Hospital of Guizhou Medical University. No patients received preoperative chemotherapy, radiotherapy, biological treatment, or Chinese medicine treatment. Also, 180-point human PC tissue microarrays were obtained from Shanghai Outdo Biotech (China).

This work was authorized by the Human Research Ethics Committee of the Affiliated Hospital of Guizhou Medical University. In addition, all patient signed an informed consent form.

### 5.3 Cell culture and transfection

AsPC-1, BxPC-3, Capan-2, Mia PaCa-2, PANC-1, and SW1990 cells were obtained from American Type Culture Collection (ATCC; USA), AsPC-1, BxPC-3, Capan-2, SW1990, and HPDE cell lines were cultured in RPMI 1640 (Gibco, USA), while Mia PaCa-2 and PANC-1 cell lines were grown in DMEM (Gibco, USA). Both medium were supplemented with 10%FBS and 1%Penicillin/Streptomycin. All cell lines were cultured in a humidified atmosphere containing 5%CO2/95% air at 37°C. All the cell lines had been authenticated through STR profiling and confirmed to be mycoplasma-free.

Lipofectamine3000 was purchased from Invitrogen (Invitrogen, USA); 3 small interfering RNAs (siRNA) (st-h-MINDY2-1 GCACAAGCCTCTCCATCAA, st-h-MINDY2-2 GCTGAGCAGTTTCTAAATA, and st-h-MINDY2-3 GTTCGAGTGTTTGAATATA) were provided by RiboBio (China). GeneChem (China) was responsible for designing and manufacturing lentivirus carrying negative control, MINDY2 overexpression vector (Ubi-MCS-3FLAG-SV40-puromycin), MINDY2-encoding short hairpin RNA (shRNA)(hU6-MCS-CMV-Puromycin), and shRNA targeting ACTN4(hU6-MCS-CMV-Puromycin). The directions were strictly followed during every infection or transfection step.

### 5.4 RNA preparation and quantitative real-time PCR

RNA was extracted from PC cell lines and PC tissue using Trizol reagent (Invitrogen, CA, USA) according to the manufacturer’s instructions. cDNA was generated by reverse transcription and used in subsequent experiments. Amplification of the generated cDNA was detected using TB Green^®^ Premix Ex TaqTM (Takara, Japan) on a CFX96TM real-time system (Bio-Rad, California, USA). The primers used in the study were: MINDY2, sense 5′- CAGGAGGCATTGCTGATGAT-3′, antisense 3′-GAAGCCTGGGGCTCATTT-5′; ACTN4, sense 5′-CACAGTCCCATTCCTCCAC-3′, antisense 3′-GCCAACCCACAAAGAGAGA-5′. GAPDH (glyceraldehyde e-3-phosphate dehydrogenase), sense 5′- CAGGAGGCATTGCTGATGAT-3′, antisense 3′-GAAGCCTGGGGCTCATTT-5′, with GAPDH as the endogenous control, and experimental The results were calculated using the 2-ΔΔCt method.

### 5.5 Antibodies and chemicals

Anti-FAM63B (1:500; ThermoFisher, # 62318), Anti-ACTN4 (1:5,000; Proteintech, #66628), Anti-GHPDH (1:1,000; Proteintech, #60004), anti-E-calmodulin (1:1,000; Proteintech, #20874), anti-N-calmodulin (1:1000; Proteintech, #22018), anti-wavoprotein (1:1,000; Proteintech, #10366), anti-snail (1:1000; Proteintech, #13099), anti-cyclin D1 (1:1,000; Cell Signaling Technology [CST], #55506), anti-cyclin E1 (1:1,000; CST, #4136), anti-CDK 2 (1:1,000; CST, #2561), anti-CDK4 (1:1,000; CST, #12790), anti-AKT (1:1,000; CST, #4691), anti-p-AKT (1:2,000; CST, #4060), anti-PI3K (1:1,000; CST, #4249), anti-p-PI3K (1:1,000; CST, #17366), anti-mTOR (1:1,000; CST, #2972), anti-p-mTOR (1: 1000; CST, #2971), HRP-goat anti-rabbit IgG (Boster, #BA1055), HRP-goat anti-mouse IgG (Boster, #BA1050), Anti-PCNA (1:1,000; Proteintech, # 10205), Anti-Ki67 (1:1,000; Proteintech, #28074), HA-Ubiquitin plasmid (Sangon Biotech, China), CHX (Melun Biologics, China), MG132 (MCE, USA), MINDY2 small interfering RNA (RiboBio, China), protease inhibitor (Boster Biological Technology, China), and enhanced chemiluminescence reagent (Proteintech, #7003).

### 5.6 Western blot

Cells were prepared and lysed with protease inhibitor-spiked RIPA buffer (Pierce). Proteins (concentration determined using a BCA assay kit (Beyotime Biotechnology)) were denatured and separated by SDS-PAGE and transferred to polyvinylidene fluoride membranes (Millipore, USA) and incubated with primary antibody at 4°C overnight and then secondary antibody at room temperature for 2h. Enhanced chemiluminescence reagents were used to detect the immunoreactive signal.

### 5.7 Cell viability assay and Colony-formation assay

At the given time points, cells were tested with Cell Counting Kit-8 (Dojindo, Japan), and absorbance at 450 nm was recorded. Cells were cultivated at 5000 cells per well in 96-well plates.

Cells were inoculated in six-well plates at 1 × 103 cells/well and cultured and cultivated for 14 days. Cells were then fixed in paraformaldehyde (4%) and stained with crystal violet (0.25 percent). The colonies were tallied and photographed.

### 5.8 Flow cytometry

PC cells were inoculated into six-well plates for 24 hours, extracted and washed with PBS, incubated for 30 minutes at room temperature with DNA staining and permeabilization solution (Cell Cycle Staining Kit, MULTI SCIENCES, China), and then protected from light. The analysis was conducted using Summit 5.2 (Beckman Coulter, USA).

### 5.9 Wound healing assay

Cells were inoculated in 6-well plates. When cell confluence reached 90-100%, the cell layer was scratched using the tip of a 200µl pipette. Cells were then incubated in a medium without FBS. The remaining distance at different time points was measured.

### 5.10 Transwell migration and Matrigel invasion assays

The Transwell device (CoStar, USA) was prepared. Then, 1×104 cells in 200 ul of FBS-free medium were plated in the upper chamber of the transwell (with or without Matrigel gel), while 800 μl of medium (containing 10% FBS) was added to the lower chamber. Cells were then incubated at 37°C in an incubator containing 5% CO2 for 24-36 hours. Next, the upper chamber was washed with PBS, and the remaining cells from the upper chamber were removed with a cotton swab. Cells in the lower chamber were fixed, tread with methanol, stained with Giemsa, dried, and counted.

### 5.11 Co-immunoprecipitation

Cells were lysed with cell lysate solution (NP-40: broad-spectrum protease inhibitor: broad-spectrum phosphatase inhibitor: PMSF = 100:2:2:1) for 30 min, centrifuged, pre-purified, and slowly shaken overnight at 4°C with the corresponding antibody. Samples were then incubated with proteinA+G, recovered the magnetic bead coupling complex wash, mixed with 2× loading buffer, and boiled. Next, subsequent Western blotting experiments were carried out.

### 5.12 Ubiquitination assay

The ubiquitinated plasmid (purchased from Sangon Biotech) was used to transfect the cells by Lipo Lipofectamine3000 following the manufacturer’s instructions. Cells were treated with the proteasome inhibitor MG132 for 9 hours and 2 days later. Then, they were removed in preparation for IP and WB assays.

### 5.13 Animal study

Female BALB/c nude mice, aged 6-7 weeks, were obtained from Collective Pharmachem. All the animals were housed in an environment with a temperature of 22 ± 1 °C, relative humidity of 50 ± 1%, and a light/dark cycle of 12/12 hr. All animal studies (including the mice euthanasia procedure) were done in compliance with the regulations and guidelines of Guizhou Medical University’s institutional animal care and conducted according to the AAALAC and the IACUC guidelines.

Mice received a subcutaneous injection of 2x106 cells (BxPC-3 and PANC-1) into the right axilla and were then randomly divided into 5 groups (5 mice/group). Tumor volume was measured periodically using vernier calipers, computed as (length × width2)/2. After five weeks, nude mice were executed and tumors were extracted, weighed, photographed, and sectioned for additional research. Liver metastasis model construction: Nude mice were randomly divided into 5 groups (n=5), BxPC-3 and PANC-1 were adjusted to 1×106 cell density according to the corresponding groups, and then 200 μL of cell suspension was injected into the spleen of nude mice, after 6 weeks, the nude mice were observed with bioluminescence and photographed the liver metastases under the small animal in the Vivo imaging system (IVIS^®^ Lumina III). After the photographs were taken, the nude mice were sacrificed, and the liver was taken for HE staining.

### 5.14 Immunohistochemistry

Fresh tumor tissue was fixed and kept at 4°C overnight, paraffin-embedded, and sectioned. It was then incubated with antibody overnight at 4°C, samples were treated with AEC chromogenic substrate, followed by hematoxylin re-staining and microscopic observation. The results were evaluated blindly by two independent pathologists.

### 5.15 Statistical analysis

Continuous data are expressed as mean ± standard deviation. Group pairs and multiple groups were compared using a two-tailed Student t-test and one-way ANOVA. Data analysis was carried out using GraphPad Prism 8.0. Image examination was done using ImageJ V1.46. A p-value <0.05 was considered to be statistically significant.

## Data availability statement

The datasets presented in this study can be found in online repositories. The names of the repository/repositories and accession number(s) can be found in the article/**Supplementary Material**.

## Ethics statement

The studies involving human participants were reviewed and approved by The Ethics Committee of the Affiliated Hospital of Guizhou Medical University. The patients/participants provided their written informed consent to participate in this study. The animal study was reviewed and approved by The Animal Care Welfare Committee of Guizhou Medical University. Written informed consent was obtained from the individual(s) for the publication of any potentially identifiable images or data included in this article.

## Author contributions

PL, SL, and CZ designed the experiments and performed most of the experiments; YoL , YiL analyzed data and helped with mice model construction; XF, XW, and JH revised this manuscript, XW and YP conceptualized the research and directed the study. All authors read and approved the final manuscript.
