# Supplementary figures and images for: The deubiquitinating enzyme MINDY2 promotes pancreatic cancer proliferation and metastasis by stabilizing ACTN4 expression and activating the PI3K/AKT/mTOR signaling pathway
Source: Front Oncol. 2023 May 3;13:1169833. doi: 10.3389/fonc.2023.1169833 (PMC10189038; doi:10.3389/fonc.2023.1169833)

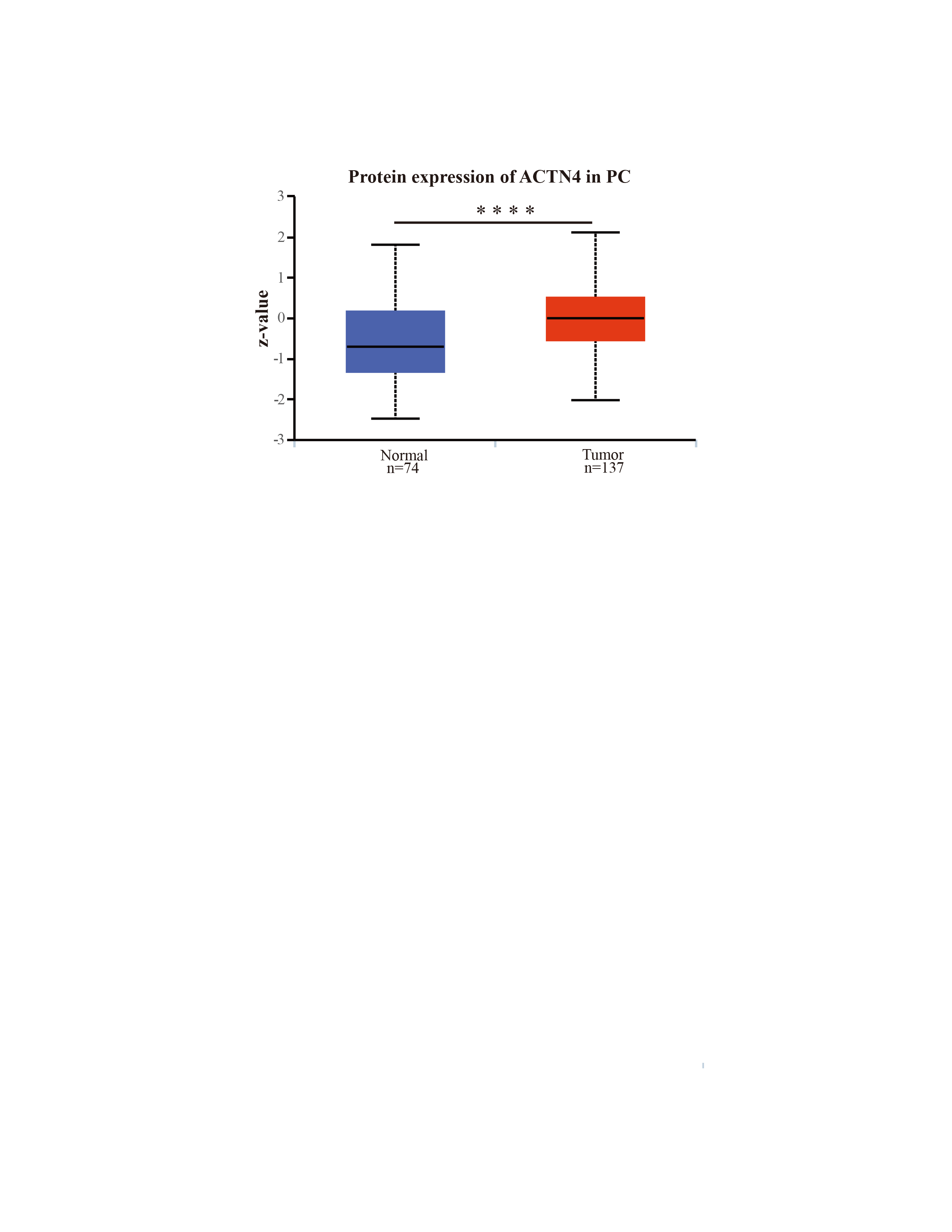

Supplement: Supplementary file 1 [file Image_1.tif]
